# 565 Pediatric Patients with Post-Burn Amputations Report Worse Long-Term Physical Function but Not Self Appearance

**DOI:** 10.1093/jbcr/irae036.199

**Published:** 2024-04-17

**Authors:** Deborah Choe, Andrew Humbert, Erin Wolfe, Sarah A Stoycos, Samuel P Mandell, Barclay T Stewart, Gretchen J Carrougher, Karen J Kowalske, Jeffrey C Schneider, David M Crandell, Haig A Yenikomshian

**Affiliations:** Keck School of Medicine of USC, Los Angeles, CA; University of Washington, Seattle, WA; University of Southern California, Los Angeles, CA; Keck School of Medicine, University of Southern California, Los Angeles, CA; UT Southwestern / Parkland Trauma, Dallas, TX; UW Medicine Regional Burn Center, Seattle, WA; UT Southwestern Medical Center, Dallas, Dallas, TX; Spaulding Rehabilitation Hospital/Harvard Medical School, Boston, MA; Spaulding Rehabilitation Hospital, Harvard Medical School Dept of PM&R, Charlestown, MA; Keck School of Medicine of USC, Los Angeles, CA; University of Washington, Seattle, WA; University of Southern California, Los Angeles, CA; Keck School of Medicine, University of Southern California, Los Angeles, CA; UT Southwestern / Parkland Trauma, Dallas, TX; UW Medicine Regional Burn Center, Seattle, WA; UT Southwestern Medical Center, Dallas, Dallas, TX; Spaulding Rehabilitation Hospital/Harvard Medical School, Boston, MA; Spaulding Rehabilitation Hospital, Harvard Medical School Dept of PM&R, Charlestown, MA; Keck School of Medicine of USC, Los Angeles, CA; University of Washington, Seattle, WA; University of Southern California, Los Angeles, CA; Keck School of Medicine, University of Southern California, Los Angeles, CA; UT Southwestern / Parkland Trauma, Dallas, TX; UW Medicine Regional Burn Center, Seattle, WA; UT Southwestern Medical Center, Dallas, Dallas, TX; Spaulding Rehabilitation Hospital/Harvard Medical School, Boston, MA; Spaulding Rehabilitation Hospital, Harvard Medical School Dept of PM&R, Charlestown, MA; Keck School of Medicine of USC, Los Angeles, CA; University of Washington, Seattle, WA; University of Southern California, Los Angeles, CA; Keck School of Medicine, University of Southern California, Los Angeles, CA; UT Southwestern / Parkland Trauma, Dallas, TX; UW Medicine Regional Burn Center, Seattle, WA; UT Southwestern Medical Center, Dallas, Dallas, TX; Spaulding Rehabilitation Hospital/Harvard Medical School, Boston, MA; Spaulding Rehabilitation Hospital, Harvard Medical School Dept of PM&R, Charlestown, MA; Keck School of Medicine of USC, Los Angeles, CA; University of Washington, Seattle, WA; University of Southern California, Los Angeles, CA; Keck School of Medicine, University of Southern California, Los Angeles, CA; UT Southwestern / Parkland Trauma, Dallas, TX; UW Medicine Regional Burn Center, Seattle, WA; UT Southwestern Medical Center, Dallas, Dallas, TX; Spaulding Rehabilitation Hospital/Harvard Medical School, Boston, MA; Spaulding Rehabilitation Hospital, Harvard Medical School Dept of PM&R, Charlestown, MA; Keck School of Medicine of USC, Los Angeles, CA; University of Washington, Seattle, WA; University of Southern California, Los Angeles, CA; Keck School of Medicine, University of Southern California, Los Angeles, CA; UT Southwestern / Parkland Trauma, Dallas, TX; UW Medicine Regional Burn Center, Seattle, WA; UT Southwestern Medical Center, Dallas, Dallas, TX; Spaulding Rehabilitation Hospital/Harvard Medical School, Boston, MA; Spaulding Rehabilitation Hospital, Harvard Medical School Dept of PM&R, Charlestown, MA; Keck School of Medicine of USC, Los Angeles, CA; University of Washington, Seattle, WA; University of Southern California, Los Angeles, CA; Keck School of Medicine, University of Southern California, Los Angeles, CA; UT Southwestern / Parkland Trauma, Dallas, TX; UW Medicine Regional Burn Center, Seattle, WA; UT Southwestern Medical Center, Dallas, Dallas, TX; Spaulding Rehabilitation Hospital/Harvard Medical School, Boston, MA; Spaulding Rehabilitation Hospital, Harvard Medical School Dept of PM&R, Charlestown, MA; Keck School of Medicine of USC, Los Angeles, CA; University of Washington, Seattle, WA; University of Southern California, Los Angeles, CA; Keck School of Medicine, University of Southern California, Los Angeles, CA; UT Southwestern / Parkland Trauma, Dallas, TX; UW Medicine Regional Burn Center, Seattle, WA; UT Southwestern Medical Center, Dallas, Dallas, TX; Spaulding Rehabilitation Hospital/Harvard Medical School, Boston, MA; Spaulding Rehabilitation Hospital, Harvard Medical School Dept of PM&R, Charlestown, MA; Keck School of Medicine of USC, Los Angeles, CA; University of Washington, Seattle, WA; University of Southern California, Los Angeles, CA; Keck School of Medicine, University of Southern California, Los Angeles, CA; UT Southwestern / Parkland Trauma, Dallas, TX; UW Medicine Regional Burn Center, Seattle, WA; UT Southwestern Medical Center, Dallas, Dallas, TX; Spaulding Rehabilitation Hospital/Harvard Medical School, Boston, MA; Spaulding Rehabilitation Hospital, Harvard Medical School Dept of PM&R, Charlestown, MA; Keck School of Medicine of USC, Los Angeles, CA; University of Washington, Seattle, WA; University of Southern California, Los Angeles, CA; Keck School of Medicine, University of Southern California, Los Angeles, CA; UT Southwestern / Parkland Trauma, Dallas, TX; UW Medicine Regional Burn Center, Seattle, WA; UT Southwestern Medical Center, Dallas, Dallas, TX; Spaulding Rehabilitation Hospital/Harvard Medical School, Boston, MA; Spaulding Rehabilitation Hospital, Harvard Medical School Dept of PM&R, Charlestown, MA; Keck School of Medicine of USC, Los Angeles, CA; University of Washington, Seattle, WA; University of Southern California, Los Angeles, CA; Keck School of Medicine, University of Southern California, Los Angeles, CA; UT Southwestern / Parkland Trauma, Dallas, TX; UW Medicine Regional Burn Center, Seattle, WA; UT Southwestern Medical Center, Dallas, Dallas, TX; Spaulding Rehabilitation Hospital/Harvard Medical School, Boston, MA; Spaulding Rehabilitation Hospital, Harvard Medical School Dept of PM&R, Charlestown, MA

## Abstract

**Introduction:**

Amputation after burn injury may improve survival rates; however, the physical changes and functional impairments resulting from amputation can have long-term consequences. A prior Burn Model System (BMS) national database study found that post-burn amputation among adults was negatively correlated with mental health scores but positively correlated with physical function scores at 6-months post-burn. However, no study has examined long-term outcomes associated with post-burn amputation in the pediatric population. This study investigates longitudinal functional and psychosocial outcomes among pediatric burn patients with amputations.

**Methods:**

Pediatric participants (8 – 17 years old) enrolled in the BMS database between 2015 – 2023 with post-burn amputations were included. Participants with amputations were matched using nearest-neighbor matching to those without, based on burn location, age and % total burn surface area. Primary outcomes were the PROMIS-25 v2.0 Physical Function and the Child Burn Outcomes Questionnaire: appearance sub-score, measured at 6-, 12- and 24-months post-burn. In addition to cross-sectional pairwise analyses at each time-point, linear mixed effects models were used to assess differences in outcomes between those with and those without amputations after adjusting for time while accounting for repeated measures. Additional linear mixed effects models, including an interaction between amputation status and time, were conducted to assess whether the change in outcome scores across time differed between those with and without amputations. A two-sided significance level of 0.05 was used to determine statistical significance.

**Results:**

In this study, 17 participants had amputations and 17 did not. A total of 7 participants had upper amputation only, 1 had lower amputation only, 2 had upper and lower amputation and 7 were unknown. Amputation was significantly associated with lower physical function scores (9.3 points lower, p=0.006) after adjusting for follow-up time-points. However, there was no evidence that the difference in physical function scores worsened or improved over time. Pairwise analyses at each time-point found those with amputations reported significantly lower physical function scores at 24-months post-burn (54.9 ±11.6 vs. 66 ±5, p=0.013). No significant differences were found in appearance scores or in the change in appearance scores.

**Conclusions:**

Amputation was significantly associated with worse physical function scores. Participants with amputation fared as well in appearance scores as those without amputation. Pediatric burn patients with amputations might benefit from tailored amputee-specific rehabilitation services, amputee peer support, and school reentry programs.

**Applicability of Research to Practice:**

This study can be used to guide recommendations for physical/occupational therapy and other rehabilitative services available to pediatric burn patients with amputations.

